# Influencing factors and profile differences in death attitudes of advance care planning readiness among patients with chronic heart failure: a latent profile analysis

**DOI:** 10.3389/fpubh.2026.1825995

**Published:** 2026-04-29

**Authors:** Jian Lin, Mengying Yang, Ruijia Xu, Yunchao Deng, Ting Huang

**Affiliations:** 1 Department of Cardiovascular Medicine, The Central Hospital of Wuhan, Tongji Medical College, Huazhong University of Science and Technology, Wuhan, China; 2 Key Laboratory for Molecular Diagnosis of Hubei Province, The Central Hospital of Wuhan, Tongji Medical College, Huazhong University of Science and Technology, Wuhan, China

**Keywords:** advance care planning, chronic heart failure, death attitudes, influencing factors, latent profile analysis

## Abstract

**Objective:**

A cross-sectional study was conducted in a tertiary A-level general hospital in Wuhan, China. The study aimed to explore the latent profiles of advance care planning (ACP) readiness among patients with chronic heart failure (CHF), analyze the influencing factors of different profiles, and compare the differences in death attitudes between profiles.

**Methods:**

From February to December 2025, a total of 313 CHF patients were recruited by convenience sampling from the Department of Cardiovascular Medicine, The Central Hospital of Wuhan, Tongji Medical College, Huazhong University of Science and Technology. The participants were investigated using a general information questionnaire, the ACP Readiness Scale, the Death Attitude Profile-Revised, and the Social Support Rating Scale. Latent profile analysis (LPA) was conducted using Mplus 8.3 to identify latent profiles of ACP readiness. Binary logistic regression was used to explore the influencing factors of profile membership, and the Bolck-Croon-Hagenaars (BCH) method was employed to compare differences in death attitudes between the latent profiles.

**Results:**

Two latent profiles of ACP readiness were identified: the “low ACP readiness” group (56.87%) and the “high ACP readiness” group (43.13%). Binary logistic regression showed that higher educational level, longer disease course, higher NYHA functional class, and greater social support were statistically significant positive predictors of belonging to the “high ACP readiness” group (*P* < 0.05). The BCH analysis revealed that the “low ACP readiness” group scored significantly higher on fear of death and death avoidance than the “high ACP readiness” group, while the “high ACP readiness” group scored significantly higher on natural acceptance (*P* < 0.05). No significant differences were found between the two groups in approach acceptance or escape acceptance (*P* > 0.05).

**Conclusion:**

There is significant heterogeneity in ACP readiness among CHF patients. Educational level, disease course, NYHA functional class, and social support are important factors influencing profile membership. The two profiles differ significantly in fear of death, death avoidance, and natural acceptance. Healthcare providers should develop targeted intervention strategies based on the characteristics of different profiles and strengthen death education to enhance patients' ACP readiness.

## 1 Introduction

Heart failure (HF) is a complex clinical syndrome caused by various etiologies that lead to abnormal changes in cardiac structure and/or function, thereby inducing ventricular systolic and/or diastolic dysfunction. It represents the severe manifestation or advanced stage of various heart diseases, with persistently high mortality and rehospitalization rates (1). According to the Global Burden of Disease (GBD) 2021 study, the global burden of HF remains substantial and continues to rise. In 2021, the total number of people living with HF worldwide reached 55.50 million. From 1990 to 2021, the global number of HF cases increased by 118.21%, with a further 33.28% increase observed between 2010 and 2021 (2, 3). According to Chinese survey data from 2012 to 2015, the prevalence of HF among adults aged ≥35 years was 1.3%, corresponding to approximately 13.7 million HF patients, an increase of 0.4% compared with 2000 (1).

In the advanced stage of chronic heart failure (CHF), patients often face unpredictable disease trajectories and heavy symptom burden, leading to a significant decline in quality of life (4). Such symptom burden and uncertainty about prognosis often result in inconsistencies between care decisions and patients' wishes, exacerbating the suffering of patients at the end of life (5, 6). Studies have shown that in terms of end-of-life care wishes, medical staff usually have difficulty achieving effective communication with terminally ill patients and their families (7), nor can they accurately predict patients' choices regarding life-sustaining treatment (8). This dilemma further highlights the importance of clarifying patients' care wishes in advance.

Advance Care Planning (ACP) refers to the process in which patients, when conscious and capable of making decisions, pre-specify their treatment and care wishes when they enter the terminal state, and communicate these wishes with medical staff and their families (9). Multiple studies have confirmed that the implementation of ACP can effectively protect patients' autonomy, uphold their dignity at the end of life, reduce decision-making conflicts, further improve the quality of life of patients at the end of life, reduce excessive medical treatment, and alleviate the decision-making distress of their families (10, 11).

It is worth noting that good ACP readiness is a key prerequisite for predicting whether patients are willing to participate in and implement ACP in the future (12, 13). Accurately assessing patients' ACP readiness level can not only clarify the feasibility of ACP implementation but also provide direction for the subsequent development of intervention measures tailored to individual patients' needs (14, 15). However, current relevant studies mostly rely on total scale scores to evaluate ACP readiness and its potential influencing factors among CHF patients. This variable-centered assessment method is difficult to fully capture the heterogeneous characteristics among individuals, which may lead to a lack of pertinence in the subsequently developed intervention measures and inability to meet the actual needs of patients with different readiness levels.

Latent Profile Analysis (LPA), as an individual-centered classification technique, can better distinguish the heterogeneity among individuals and help formulate more individualized intervention methods (16). Therefore, this study used LPA to explore the different latent profiles of ACP readiness among CHF patients, combined univariate analysis and binary logistic regression analysis to identify the influencing factors related to different profiles, and used the Bolck-Croon-Hagenaars (BCH) method to compare differences in death attitudes between the latent profiles. Specifically, this study aimed to answer three core questions: whether ACP readiness among CHF patients presented significant heterogeneity and could be divided into how many clinically interpretable latent profiles; what sociodemographic and disease-related factors predicted the latent profile membership of ACP readiness; and whether patients in different ACP readiness profiles differed significantly in all five dimensions of death attitudes. The findings aim to provide a scientific basis for the effective implementation of ACP and the design of targeted interventions.

## 2 Methods

### 2.1 Study design and participants

This study adopted a cross-sectional design and was conducted in the Department of Cardiovascular Medicine, The Central Hospital of Wuhan, Tongji Medical College, Huazhong University of Science and Technology from February to December 2025. This hospital is a tertiary A-level general hospital in Wuhan, Hubei Province, China.

Convenience sampling was used to recruit eligible hospitalized patients. All potential participants were screened by uniformly trained researchers within 24–48 h of admission. The researchers first reviewed the electronic medical records to verify the diagnosis of CHF and NYHA functional class, then explained the study purpose, procedures, risks and benefits to eligible patients face-to-face. Patients who voluntarily agreed to participate signed the written informed consent form before data collection.

The inclusion criteria for participation in this study were as follows: (1) individuals aged ≥ 18 years; (2) individuals diagnosed with CHF based on the 2024 Chinese guidelines for the diagnosis and treatment of heart failure; (3) individuals with New York Heart Association functional class II to IV; (4) individuals who provided informed consent and voluntarily participated in the study. Exclusion criteria were as follows: (1) individuals with cognitive impairment; (2) individuals complicated with severe organ dysfunction or other serious diseases (e.g., malignant tumors).

The sample size was estimated to be 10 to 15 times the number of independent variables. A total of 16 independent variables were included in this study. Considering a 20% rate of invalid questionnaires, the minimum required sample size was calculated to be at least 200 participants.

### 2.2 Ethical approval

This study was conducted in strict accordance with the ethical principles proposed in the Declaration of Helsinki. All participating patients provided informed consent after being fully informed of the study purpose and procedures. Meanwhile, this study was approved by the Ethics Committee of the Central Hospital of Wuhan (Approval No.: WHZXKYL2022-205-02).

### 2.3 Measures

#### 2.3.1 General information questionnaire

This questionnaire was independently developed by the research team after reading relevant literature and consulting experts. It includes items such as gender, age, educational level, marital status, religious belief, place of residence, family per capita monthly income, disease duration, and NYHA functional class, etc.

#### 2.3.2 Advance care planning readiness scale (ACPRS)

This scale was developed by Wang and Sheng (17) on the basis of literature review, patient interviews, and expert consultation. It is an assessment tool for the readiness of ACP in chronically ill patients that conforms to the Chinese cultural context. The scale consists of 3 dimensions with 22 items: Attitude toward ACP (10 items), Belief in participating in ACP (7 items), and Motivation to participate in ACP (5 items). A 5-point Likert scale is adopted, ranging from “strongly disagree” to “strongly agree,” with scores from 1 to 5 in turn, and the Attitude dimension is scored in reverse. The Cronbach's α coefficient of the scale is 0.923, the content validity is 0.986, and the Cronbach's α coefficients of the 3 dimensions are all above 0.835. The higher the score, the better the ACP readiness. The full score of ACP readiness ranges from 22 to 110, and the total score can be divided into 4 levels: 22–43 points for low level, 44-65 points for below-average level, 66–87 points for above-average level, and 88–110 points for high level.

#### 2.3.3 Death attitude profile-revised (DAP-R)

This scale was developed by Wong et al. (18) in 1994, and culturally adapted by mainland Chinese scholars Tang et al. (19); it consists of 5 dimensions with 32 items, evaluating five types of individual attitudes toward death respectively, including fear of death, death avoidance, natural acceptance, approach acceptance, and escape acceptance. Each item is scored on a 5-point Likert scale, with 1 point for “Strongly Disagree” and 5 points for “Strongly Agree,” and the total score of the scale is not calculated, but instead the scores of each subscale are compared for interpretation, where a higher score of a subscale indicates that the individual's attitude toward death is more inclined to that dimension, and the Cronbach's α coefficient of the Chinese version of the scale is 0.875.

#### 2.3.4 Perceived social support scale (PSSS)

This scale was developed by Zimet et al. (20), sinicized by Chinese scholar Jiang (21), and is used to investigate the level of social support perceived by patients from different groups. It consists of 3 dimensions including family support, friend support, and other support, with a total of 12 items. Each item is scored on a 7-point Likert scale, ranging from 1 point for “Strongly Disagree” to 7 points for “Strongly Agree,” and the total score ranges from 12 to 84 points. A higher score indicates a higher level of social support perceived by the patient.

### 2.4 Data collection and quality control

Prior to this study, all investigators received unified training on research purpose, questionnaire standards, instruction requirements and quality control points to ensure consistent operations. During the study, standardized paper questionnaires were administered to subjects via one-to-one interviews. Before formal data collection, investigators read standardized instructions to patients, explaining the research purpose, filling methods, questionnaire scope and precautions to protect their right to informed consent. Questionnaires were distributed only after patients fully understood, volunteered to participate and signed the informed consent form. For patients unable to complete the questionnaire independently due to educational level or physical conditions, explicit consent was obtained from themselves and their family members (if applicable). Investigators then filled out the questionnaire truthfully based on patients' verbal responses without guidance or hints to ensure authenticity. Patients' general disease-related data were accurately completed by reviewing the hospital's electronic medical records, followed by a recheck to avoid omissions or entry errors. Questionnaires were distributed and collected on-site. Upon collection, investigators checked for missing or non-standard items, excluding invalid ones with patterned responses (e.g., consecutive same selections). In the data entry stage, two independent investigators separately entered valid questionnaire data into the designated database. The two datasets were cross-checked to identify and correct entry errors, ensuring data accuracy and reliability.

### 2.5 Statistical methods

Data processing and latent profile modeling were conducted using the Mplus 8.3 software package. Starting from the baseline model with one latent profile, the number of latent classes was increased gradually, and model fit indices were tested for models with different class numbers; the optimal model was selected according to model fit statistics and clinical practicality. The main model fit indices included the Akaike information criterion (AIC), Bayesian information criterion (BIC), sample-size adjusted Bayesian information criterion (aBIC), Lo-Mendell-Rubin likelihood ratio test (LMRT), and Bootstrap-based likelihood ratio test (BLRT), while entropy was used to evaluate classification accuracy. Generally, smaller AIC, BIC, and aBIC values indicated better model fit; an entropy value closer to 1 suggested higher classification accuracy, and an entropy value ≥ 0.8 indicated a classification accuracy above 90%. Significant LMRT and BLRT values (*P* < 0.05) suggested a significant improvement in model fit compared with the model with one fewer class.

After identifying the optimal latent profile model, the BCH method was employed to examine differences in death attitudes across the identified profiles. This method accounts for classification uncertainty and compares the mean scores of continuous distal outcomes (the five dimensions of death attitudes: fear of death, death avoidance, natural acceptance, approach acceptance, and escape acceptance) between latent profiles.

Data analysis was performed using SPSS 27.0. Normally distributed measurement data were expressed as mean ± standard deviation, and between-group comparisons were conducted using the independent-samples *t*-test; non-normally distributed measurement data were expressed as the median and interquartile range, and between-group comparisons were analyzed using the Mann-Whitney U test. Enumeration data were presented as frequencies, percentages or rates, and between-group comparisons were performed using the chi-square (χ^2^) test. Finally, to explore the influencing factors associated with different latent profiles of ACP readiness, a binary logistic regression analysis was applied. The test level for statistical significance was set at α = 0.05.

## 3 Results

### 3.1 Characteristics of study participants

A total of 365 consecutive hospitalized patients with a primary diagnosis of CHF were initially screened during the study period. Among them, 35 patients did not meet the inclusion criteria (31 patients had cognitive impairment, and 4 patients had concurrent malignant tumors). The remaining 330 eligible patients were invited to participate in the survey face-to-face. Seven patients withdrew voluntarily for personal reasons, and 10 questionnaires were deemed invalid due to patterned responses (defined as 10 or more consecutive identical selections). Finally, 313 valid questionnaires were included in the final analysis, yielding an effective response rate of 94.85%. The general characteristics of the study participants are shown in Table 1. The total score of ACP readiness among CHF patients was 56 (50, 71).

**Table 1:** General information of participants and univariate analysis of the latent profile of ACP readiness (*n* = 313).

Variables	Overall	Low ACP readiness	High ACP readiness	*t*/*χ^2^*/*Z*	*P*
Age, M (P_25_, P_75_)	59 (51, 67)	58 (51, 67)	59 (50, 67)	−0.107	0.915
Gender, *N* (%)					
Male	197 (62.9%)	119 (66.9%)	78 (57.8%)	2.711	0.1
Female	116 (37.1%)	59 (33.1%)	57 (42.2%)		
Marital status, *N* (%)					
Married	184 (58.8%)	99 (55.6%)	85 (63.0%)	1.710	0.191
Unmarried/Divorced/Widowed	129 (41.2%)	79 (44.4%)	50 (37.0%)		
Educational level, N(%)					
Junior high school and below	80 (25.6%)	62 (34.8%)	18 (13.3%)	−3.980	<0.001
Senior high school/Technical secondary school	114 (36.4%)	60 (33.7%)	54 (40.0%)		
Junior college and above	119 (38.0%)	56 (31.5%)	63 (46.7%)		
Religious belief, *N* (%)					
No	257 (82.1%)	152 (85.4%)	105 (77.8%)	3.031	0.082
Yes	56 (17.9%)	26 (14.6%)	30 (22.2%)		
Place of residence, *N* (%)					
Rural area	102 (32.6%)	58 (32.6%)	44 (32.6%)	0.356	0.837
County town	107 (34.2%)	63 (35.4%)	44 (32.6%)		
City	104 (33.2%)	57 (32.0%)	47 (34.8%)		
Family per capita monthly income, *N* (%)					
<3,000	119 (38.0%)	65 (36.5%)	54 (40.0%)	−1.200	0.230
3,000–5,000	96 (30.7%)	51 (28.7%)	45 (33.3%)		
>5000	98 (31.3%)	62 (34.8%)	36 (26.7%)		
Disease course, *N* (%)					
<2 years	100 (31.9%)	79 (44.4%)	21 (15.6%)	−5.822	<0.001
2–5 years	92 (29.4%)	51 (28.7%)	41 (30.4%)		
>5 years	121 (38.7%)	48 (27.0%)	73 (54.1%)		
NYHA functional class, *N* (%)				−6.226	<0.001
II	140 (44.7%)	107 (60.1%)	33 (24.4%)		
III	130 (41.5%)	56 (31.5%)	74 (54.8%)		
IV	43 (13.7%)	15 (8.4%)	28 (20.7%)		
The experience of caring for the family members of the terminally ill, *N* (%)					
No	158 (50.5%)	86 (48.3%)	72 (53.3%)	0.774	0.379
Yes	155 (49.5%)	92 (51.7%)	63 (46.7%)		
Social support, M(P_25_, P_75_)/Mean ± SD	53 (49, 57)	50.13 ± 4.84	57.21 ± 4.72	−12.951	<0.001

A higher NYHA functional class indicates poorer cardiac function.

### 3.2 LPA of ACP readiness among CHF patients

In this study, 1 to 3 latent profile models were sequentially fitted to explore the ACP readiness of CHF patients. The results showed that the AIC, BIC, and aBIC values decreased continuously as the number of classes increased. The LMRT of the 3-class model was not statistically significant (*P* > 0.05), whereas the entropy value was the highest in the 2-class model. Therefore, the 2-class model was determined to be the optimal fitting model. The fitting indices are presented in Table 2.

**Table 2:** Model fit indices of the latent profile analysis for ACP readiness among CHF patients (*n* = 313).

Model	AIC	BIC	aBIC	Entropy	*P*(LMRT)	*P*(BLRT)	Latent profile probability (%)
1	1,870.620	1,893.098	1,874.068	—	—	—	100
2	1,445.213	1,482.675	1,450.958	0.940	<0.001	<0.001	56.87/43.13
3	1,398.273	1,450.720	1,406.316	0.941	0.0572	<0.001	5.11/57.83/37.06

AIC, Akaike Information Criterion; BIC, Bayesian Information Criterion; aBIC, sample-size adjusted Bayesian Information Criterion; LMRT, Lo-Mendell-Rubin likelihood ratio test; BLRT, Bootstrap-based Likelihood Ratio Test; –indicates no data available for this item.

### 3.3 Profile characteristics and naming of ACP readiness among CHF patients

**Figure 1:**
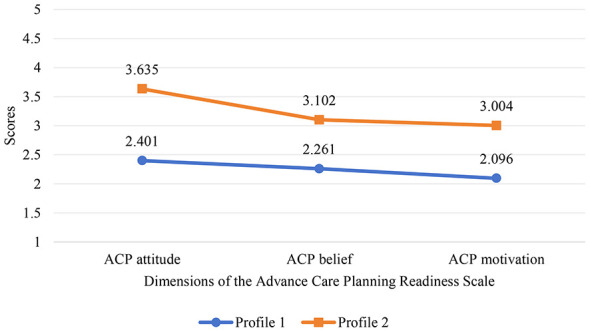
Profile plot of the two latent profiles of ACP readiness among CHF patients.

Based on the optimal 2-class model, a latent profile plot was constructed with the three dimensions of the ACP Readiness Scale (ACP attitude, ACP belief, and ACP motivation) as the horizontal axis and the mean scores of each dimension as the vertical axis (Figure 1). Profile 1 included 178 participants (56.87%), who showed lower mean scores across all three dimensions. Accordingly, this profile was named the “low ACP readiness.” Profile 2 comprised 135 participants (43.13%), who exhibited higher mean scores across all three dimensions. This pattern indicated more positive attitudes and greater acceptance of ACP. Thus, this profile was labeled the “high ACP readiness.”

### 3.4 Univariate analysis of latent profile of ACP readiness

Univariate analysis revealed statistically significant differences between the two latent profiles in terms of educational level, disease course, NYHA functional class, and social support (*P* < 0.05). See Table 1.

### 3.5 Binary logistic regression analysis of latent profile of ACP readiness

Binary logistic regression analysis was performed with the two latent profiles of ACP readiness as the dependent variable (with the “low ACP readiness” group as the reference) and the variables that were statistically significant in the univariate analysis as independent variables. The assignments of independent variables are shown in Table 3, and the results of the regression analysis are presented in Table 4. The results showed that higher educational level, longer disease course, higher NYHA functional class, and greater social support were statistically significant positive predictors of belonging to the “high ACP readiness” group (*P* < 0.05). The model demonstrated good fit (Hosmer-Lemeshow test: **χ^2^** = 6.477, *P* = 0.594).

**Table 3:** Assignment of independent variables.

Variables	Assignment method
Educational level	Junior high school and below = 1; Senior high school/Technical secondary school = 2; Junior college and above = 3
Disease course	<2 years = 1; 2–5 years = 2; >5 years = 3
NYHA functional class	II = 1; III = 2; IV = 3
Social support	Original value input

**Table 4:** Binary logistic regression analysis of latent profile of ACP readiness.

Variables	β	Standard error	*P*	*OR*	95% *CI*
Educational level	0.419	0.211	0.047	1.520	1.005–2.300
Disease course	1.088	0.208	<0.001	2.968	1.976–4.457
NYHA functional class	0.717	0.229	0.002	2.048	1.308–3.207
Social support	0.311	0.040	<0.001	1.365	1.261–1.476

### 3.6 Comparisons of death attitudes between two latent profiles

BCH method was used to compare differences in death attitudes between the two latent profiles. Patients in the “low ACP readiness” group scored significantly higher on fear of death and death avoidance than those in the “high ACP readiness” group, whereas patients in the “high ACP readiness” group scored significantly higher on natural acceptance (*P* < 0.05). No significant differences were observed between the two groups in approach acceptance or escape acceptance (*P* > 0.05). The results are presented in Table 5.

**Table 5:** Comparisons of death attitudes between two latent profiles by the BCH method.

Death attitudes	Low ACP readiness M(SE)	High ACP readiness M(SE)	*χ^2^*	*P*
Fear of death	3.046 (0.037)	2.813 (0.045)	15.640	<0.001
Death avoidance	3.177 (0.046)	2.908 (0.058)	12.878	<0.001
Natural acceptance	2.822 (0.040)	3.132 (0.054)	20.674	<0.001
Approach acceptance	2.462 (0.028)	2.510 (0.031)	1.276	0.259
Escape acceptance	2.694 (0.044)	2.728 (0.052)	0.244	0.621

BCH, Bolck-Croon-Hagenaars, used to test differences in continuous variables across latent profiles.

## 4 Discussion

### 4.1 Heterogeneity of ACP readiness among patients with CHF

A total of 313 CHF patients were investigated in this study. The total score for ACP readiness was 56 (50, 71), indicating a low-to-medium level, which is consistent with the findings of Shen and Yang (22). The results of LPA revealed that ACP readiness among patients with CHF could be categorized into two distinct profiles, demonstrating significant heterogeneity. The “low ACP readiness” group accounted for 56.87% of the total study population. The core characteristics of this group were insufficient ACP knowledge and emotional avoidance. These patients lacked an understanding of end-stage prognosis and the value of ACP, showed little motivation to participate, and some even misinterpreted ACP as “giving up treatment” and were unwilling to discuss their end-of-life care preferences. For these patients, a stepwise cognitive-emotional intervention can be implemented. This approach should first address their avoidant emotions, then clarify the core value of ACP through easy-to-understand explanations and case-based education. Combining this with education on the late-stage trajectory of CHF, encouraging family involvement in communication, and guiding patients to confront the topic and express their wishes may progressively enhance their ACP readiness. The “high ACP readiness” group comprised 43.13% of the total study population. This group was characterized by a good understanding of their illness. They clearly recognized the uncertainty of the late-stage prognosis of CHF and the value of ACP, and were willing to actively learn about the ACP process and communicate their care preferences, providing a solid foundation for participation. For these patients, motivational interventions are suitable. This can involve establishing a personalized ACP promotion file to affirm their proactive behavior. Furthermore, they can serve as role models, sharing their experiences with peers to encourage broader participation and enhance ACP readiness within the patient community.

### 4.2 Influencing factors of latent profiles of ACP readiness

#### 4.2.1 Educational level

Binary logistic regression analysis showed that patients with higher educational level were more likely to be classified into the “high ACP readiness” group. Previous studies have shown that educational level was positively correlated with ACP readiness among CHF patients (22, 23). As ACP is a relatively novel concept in China, individuals with higher education tend to have greater acceptance of new ideas and broader access to information resources (24). Patients with higher educational levels can proactively obtain disease-related information—such as treatment options and prognosis of heart failure—through books, the internet, and other channels. When they come to understand that heart failure is an incurable disease, they tend to reflect more deeply on issues such as personal values and the meaning of life, and are better psychologically prepared in advance. As a result, they are more capable of confronting death courageously in the advanced stages of the disease and accepting ACP with greater equanimity. A study has shown that health education could significantly improve ACP acceptance among older adults with chronic conditions in community settings (25). Therefore, for patients with lower educational levels, healthcare providers can introduce ACP knowledge and options through vivid visual aids and videos, thereby enhancing patients' understanding of ACP and helping them make end-of-life care decisions that align with their values and beliefs (26, 27).

#### 4.2.2 Disease course

The results of this study showed that patients with a longer disease course were more likely to be classified into the “high ACP readiness” group. A previous study has shown a positive correlation between disease course and ACP readiness (28). As a chronic progressive condition, the longer the disease course in CHF, the more frequently patients experience recurrent acute exacerbations. Over time, they gain a clearer understanding of the disease's progression patterns, treatment processes, and long-term prognosis, gradually shifting from initial fear toward a more rational acceptance of the illness. During this prolonged struggle with the disease, patients begin to confront its irreversibility and proactively contemplate their end-of-life care needs, thereby enhancing their awareness and acceptance of ACP.

#### 4.2.3 NYHA functional class

The results of this study showed that patients with higher NYHA functional class were more likely to be classified into the “high ACP readiness” group. A previous study has demonstrated that NYHA functional class was an important influencing factor of ACP acceptance in HF patients (29). Studies have shown that individuals in poorer health were more willing to engage in ACP (30, 31). A higher NYHA functional class reflects greater disease severity, often accompanied by severe symptoms such as dyspnea, fatigue, and edema, which significantly limit daily activities and substantially reduce quality of life. These patients also face elevated risks of hospital readmission and mortality. This direct experience of disease severity and poor prognosis prompts them to proactively confront death-related topics and express greater willingness to clarify their end-of-life care preferences in advance, thereby manifesting as high ACP readiness. Therefore, for patients with lower NYHA functional class, healthcare providers should, based on their specific disease characteristics, moderately enhance their awareness of disease risks through case-based education and prognosis prediction.

#### 4.2.4 Social support

The results of this study showed that patients with higher levels of social support were more likely to be classified into the “high ACP readiness” group. A previous study has indicated that family support was a significant influencing factor of ACP readiness in patients with chronic diseases, and that higher levels of family support were associated with greater ACP readiness among hospitalized older adults with chronic diseases (32). Support from family members and friends can provide patients with material resources and emotional comfort, enabling them to feel respected, supported, and understood. This effectively alleviates feelings of loneliness and fear when facing advanced illness and death, thereby enhancing their psychological security. In a favorable social support environment, patients are more willing to communicate openly with family and friends about their inner thoughts, including their end-of-life care preferences. They also feel more empowered to proactively learn about ACP-related knowledge and participate in ACP decision-making, which in turn enhances their ACP readiness. Previous studies have shown that family function is significantly positively correlated with ACP readiness, and higher social support predicts higher ACP readiness in older adults (33, 34). Therefore, in clinical practice, healthcare providers should pay attention to patients' social support status, proactively identify those with insufficient support, encourage family members and friends to provide more companionship and care, and help establish a diversified social support system for patients.

### 4.3 Differences in death attitudes between the latent profiles

This study employed BCH method to compare differences in death attitudes between the two latent profiles. The results showed that patients in the “low ACP readiness” group scored significantly higher on the dimensions of fear of death and death avoidance than those in the “high ACP readiness” group, while patients in the “high ACP readiness” group scored significantly higher on the dimension of natural acceptance. No statistically significant differences were found between the two groups in the dimensions of approach acceptance or escape acceptance. A previous study has indicated that death attitude is an important influencing factor of ACP acceptance among older adults with chronic diseases: patients who hold avoidant attitude toward death tend to have lower ACP acceptance, whereas those who hold naturally accepting attitudes toward death tend to have higher ACP acceptance (35). Influenced by traditional Chinese culture, death has long been a taboo topic (36), with most individuals holding negative attitudes of fear and avoidance—perspectives that run counter to the philosophy of ACP. Studies have shown that fear of and avoidance regarding death and end-of-life topics were major barriers to patient participation in ACP (37, 38). Therefore, it is essential to strengthen death education for CHF patients, helping them develop a correct view of life and death, enhancing their capacity to face death, and fostering recognition of the importance and necessity of ACP.

## 5 Limitations

Several limitations of this study should be acknowledged. First, this study only enrolled patients with CHF from a single region in China, which may introduce potential selection bias and limit the generalizability of the findings to other CHF populations. Second, this cross-sectional observational design can only reveal the association between ACP readiness, death attitudes and social support at a single time point, but cannot establish definitive causal relationships or longitudinal dynamic changes between variables. Third, the core indicators (death attitudes and ACP readiness) involve death and end-of-life decision-making, which are culturally sensitive topics in China. Affected by social taboos of death and social desirability bias, some patients may have concealed their true attitudes toward death during self-report scale completion, which may slightly affect the robustness of our results. Finally, no longitudinal follow-up was conducted in this study. We do not know whether patients with high ACP readiness will actually complete ACP documents in clinical practice. For future research, multi-center, large-sample prospective cohort studies should be conducted to verify the stability and generalizability of our findings, and longitudinal follow-up should be used to clarify the causal relationship between ACP readiness and death attitudes. In addition, mixed-methods research combining quantitative and qualitative designs should be adopted to break the cultural taboo of death through in-depth interviews, explore patients' real perceptions of end-of-life care and ACP, and provide evidence for targeted ACP promotion strategies in clinical practice.

## 6 Conclusion

This study identified two distinct profiles of ACP readiness among patients with CHF. Educational level, disease course, NYHA functional class, and social support emerged as significant predictors of profile membership. Furthermore, significant differences in death attitudes were observed between the two groups. These findings underscore the heterogeneity in ACP readiness among CHF patients and highlight the need for specific interventions. Approaches such as cognitive-emotional support for patients with avoidant tendencies and motivational enhancement for those with greater receptiveness, combined with death education may promote greater engagement in ACP.

## Data Availability

The original contributions presented in the study are included in the article/supplementary material, further inquiries can be directed to the corresponding author.
